# Caring for a Child With Cerebral Palsy: Cross-Sectional Study

**DOI:** 10.2196/89774

**Published:** 2026-08-20

**Authors:** Anwar B Almutairi, Zainab Jasem, Amneh Sadeq, Maraheb Alkhalidi

**Affiliations:** 1 Physical Therapy Department, Kuwait University, PO Box 5969, Safat, 13060, Kuwait, 965 24633500

**Keywords:** cerebral palsy, caregivers, self-care, satisfaction

## Abstract

**Background:**

Caregivers of children with cerebral palsy (CP) encounter unique challenges that are not common in typical families. The distress of these families starts in the diagnosis period, which is marked by a “wait and see” approach, and continues because of the lack of a clear prognosis.

**Objective:**

This study aimed to identify the level of perceived ease of care faced by families of children with CP in Kuwait and its effect on their satisfaction with daily activities.

**Methods:**

This cross-sectional study recruited caregivers of children with CP aged 5 to 16 years. The Ease of Caregiving for Children (ECC) scale, the self-care domain of the Child Engagement in Daily Life Measure (CEDLM), and the Satisfaction with Daily Occupations (SDO) questionnaire were used.

**Results:**

Fifty-three caregivers participated (mean age 39.77, SD 7.56 years; children’s mean age 8.04, SD 2.57 years; Gross Motor Function Classification System: level I, n=18; level II, n=10; level III, n=13; level IV, n=5; and level V, n=7). ECC score was significantly correlated with SDO score (*P*=.03) and the participation score in the self-care scale of the CEDLM (*P*=.001).

**Conclusions:**

Challenges of caring for a child with CP are positively correlated with caregivers’ satisfaction with daily activities and the child’s self-care abilities. Regular check-ins with caregivers and the establishment of support groups for caregivers would help with providing help sooner. Qualitative assessment is needed to explore these challenges in this region.

## Introduction

Cerebral palsy (CP) is a group of permanent, nonprogressive disorders that affect the infant and fetal brain, hindering the development of movement and posture [1,2]. Children with CP may experience impairments such as sensory disturbances, cognitive disabilities, communication limitations, behavioral disorders, epilepsy, and secondary musculoskeletal problems [2,3]. Other comorbidities reported in children with CP include pain, bladder control problems, blindness, sleep disorders, and hip displacement [3,4].

Caregivers of children with CP encounter unique challenges that are not commonly seen among caregivers in typical families. The distress of these families starts with the diagnosis period, which is marked by a “wait and see” approach, and continues because of the lack of a clear prognosis [4,5]. The exploratory journey that is required to discover the type of comorbidities children with CP exhibit is another demanding task for caregivers [6]. Later on, caregivers of children with CP are confronted by the constant challenges displayed by their child and the ever-present need to engage in problem-solving [2,4,6]. Families of children with CP also express concern about the transition to adolescence and, later, adulthood for these children and the level of independence they can achieve [7]. This roller coaster takes a toll on caregivers of children with CP as individuals and on the family unit as a whole [8-10]. In a recent systematic review exploring the lived experience of caregivers, the burden of caring for a child with CP was positively correlated with feelings of depression [8]. Other factors that were associated with increased caregiver burden in this group were self-efficacy and anxiety among caregivers [11,12]. Interestingly, despite the degree of depression and anxiety leading to burden, feelings of resilience have also been explored and were found to be moderately negatively correlated with caregiver burden [10].

Literature exploring burden has primarily highlighted the impact of caregiver-related factors on caregiver burden but has rarely included child-related factors (eg, child functional level or cognitive level) [8]. It is therefore imperative to include the child’s behavior, family function, and caregiving demand when exploring caregiver burden, given that they have been noted to be key predictors for caregivers’ well-being [13,14].

Despite the abundance of current literature that has greatly contributed to our understanding of the dynamics of the burden experienced by caregivers of children with CP, most of these studies have been conducted in high-income countries (ie, Canada, South Korea, and Spain). Although Kuwait is a high-income country, the information available in the literature on caregivers’ burden in Kuwait is scarce. Cultural differences in the region call for the need to explore these variables. Examining these variables (ie, caregiver burden) would allow for better tailoring of interventions to meet the needs of families with children with CP. Kuwait, similar to its surrounding region in the Arabian Peninsula (ie, Kingdom of Saudi Arabia and United Arab Emirates), has a cultural structure that is very different from that of the Western world. The family-centered culture and the availability of hired, live-in domestic caretakers [15] present a distinctive combination of advantages and disadvantages, which merits further exploration.

This study aimed to identify (1) the level of perceived ease of care faced by families of children with CP aged 5 to 16 years in Kuwait, (2) the child factors (demographics and level of independence) that influence this perceived level of burden, and (3) the relationship between the perceived level of burden and Satisfaction With Daily Occupations (SDO) score among caregivers of children with CP in Kuwait. The age range was chosen based on the cutoff used for children with CP in pediatric rehabilitation departments in Kuwait.

## Methods

### Ethical Considerations

This project was approved by the ethics committee at the Ministry of Health in Kuwait (2021/1865). The study adhered to the Declaration of Helsinki. Electronically signed informed consent forms were obtained from all participants.

### Design

This cross-sectional study used a web-based questionnaire to assess the level of perceived ease of care faced by families of children with CP aged 5 to 16 years. Inclusion criteria were (1) being a caregiver of at least one child aged 5 to 16 years with a documented diagnosis of CP, (2) residing in and receiving care in Kuwait (ie, working or studying), and (3) being able to communicate effectively (ie, read and write) in either Arabic or English. A purposive nonprobability sampling method was used to recruit participants. Study flyers were distributed in waiting rooms of rehabilitation clinics throughout Kuwait to inform parents of children with CP about the study. Interested parents contacted the research team. After screening for the inclusion and exclusion criteria, parents were sent an electronic form to complete.

### Procedure

Health care professionals in rehabilitation clinics around Kuwait working with developmental disorders were contacted by the research team and asked to inform their clients about the study using the study flyer. Participants who agreed to take part in the study were screened for the inclusion and exclusion criteria, then sent a link to the web-based form. Participants were asked to complete an electronic form about the demographics of the caregivers and the child with CP for whom they were providing care. Caregiver-related variables included age, gender, educational level, marital status, working hours, and the number of other caregivers. Child-related variables included age, gender, topographic type, Gross Motor Function Classification System (GMFCS) level, hand function (ie, independent, partially dependent, or totally dependent), height and weight, and schooling situation. Next, participants scored their experience using (1) the Ease of Caregiving for Children (ECC) measure, (2) participation in the self-care domain of the Child Engagement in Daily Life Measure (CEDLM), and (3) the SDO measure. The language in which each of the tools was presented was based on the participant’s preferences.

### Instruments

ECC is a self-reported tool developed to examine the physical demands of caring for a child with CP [16]. Caregivers were asked to rate their perceived level of physical demands associated with caring for children using 12 items relating to mobility, positioning, and self-care. The level of demand was rated using a 5-point Likert scale (1=“very difficult” to 5=“no help is needed”). Higher scores (maximum 60) indicate greater ease of care perceived by the caregiver (ie, less burden of caregiving) [17]. Both English and Arabic versions were reliable (intraclass correlation coefficient [ICC] >0.69) and valid [17,18]. Cultural adaptation was established in Arabic [17].

The self-care domain of the CEDLM is an 18-item caregiver-reported measure that assesses the caregivers’ perceptions of the child’s level of independence with their self-care activities (eg, toileting, dressing, feeding, drinking, and grooming) [17]. The level of independence is rated using a 5-point Likert scale (1=“does not do the activity” to 5=“does the activity independently most of the time”). A high level of independence with self-care activities is indicated by a higher score (maximum 90) [17]. Both English and Arabic versions of the scale were reliable (ICC=0.96) and valid [17,19]. Cultural adaptation was established in Arabic [17].

The SDO is a self-reported tool that was used to assess caregivers’ satisfaction with daily activities. This tool explores different daily activities where a higher score indicates a higher level of participation and satisfaction across the constructs of work (4 items), leisure (3 items), home management (4 items), and self-care (3 items) [20,21]. This 14-item tool examines level of performance (range 0‐14) and the level of satisfaction (range 14‐70) [20,21]. Reliability (ICC=0.93) and validity of both Arabic and English versions of the scale were established [20,21]. Cultural adaptation was established in Arabic [20].

### Data Analysis

The data were analyzed using SPSS Statistics for Windows (version 27.0; IBM Corp). The statistical significance level was set at α=.05 and normality of all variables was explored using the Shapiro-Wilk test. Normally distributed variables were assessed using parametric tests, whereas variables that did not follow a normal distribution were assessed using nonparametric tests. Descriptive variables were expressed as mean and SD for continuous variables and as count (n) and percentage (%) for categorical variables. BMI was calculated and presented according to the classification presented by Hurvitz et al [22].

For both the ECC and self-care domain of the CEDLM, the scaled scores (using the conversion tables) [16] were calculated. The chi-square test was used to assess whether the prevalence of the CP burden, the independence of the child, and the satisfaction of the caregiver differed across categories of sociodemographic factors. Because cutoff scores were not established for these questionnaires, a 50% cutoff was used for all the tools (scaled score of ECC ≤40=substantial burden; scaled score of CEDLM ≥50.2=independent child; score of SDO ≥56=satisfied with daily occupation). These suggested cutoffs were used to establish categorization for each tool and enhance interpretation of the tools. The established criteria enabled comparison between scales. Regarding the self-care domain of the CEDLM and ECC items, the independent 1-tailed *t* test, and Mann-Whitney *U* test were used to assess the difference in means across the questionnaire items. The relationship between the total score of each of the tools (ie, ECC, the self-care domain of the CEDLM, and SDO) was evaluated using Pearson correlation coefficients.

## Results

A total of 53 caregivers (n=40, 75.5% female) participated in this study with a mean age of 39.77 (SD 7.56) years (Table 1). The mean age of the children with CP was 8.04 (SD 2.75) years (GMFCS: 1: level I, n=18; level II, n=10; level III, n=13; level IV, n=5; and level V, n=7).; Table 2). All participants chose to use the Arabic forms of the assessment tools.

**Table 1.** Characteristics of the study sample—caregiver data^a^.

	Total sample (n=53)	Ease of Caregiving for Children			Child Engagement in Daily Life Measure			Satisfaction With Daily Occupations		
		Significant burden (n=17)	No significant burden (n=36)	*P* value^b^	Independent (n=25)	Dependent (n=28)	*P* value^b^	Yes (n=12)	No (n=41)	*P* value^b^
Sex, n (%)				.60			.41			.13
Male	13 (24.5)	4 (23.5)	9 (25)		7 (28)	6 (21.4)		1 (8.3)	12 (29.3)	
Female	40 (75.5)	13 (76.5)	27 (75)		18 (72)	22 (78.6)		11 (91.7)	29 (70.7)	
Age (years), mean (SD)	39.77 (7.56)	41.24 (7.90)	39.08 (7.32)	.34	37.76 (6.36)	41.57 (8.08)	.046	36.50 (9.10)	40.73 (6.80)	.09
Marital status, n (%)				.62			.26			.22
Married	49 (92.5)	16 (94.1)	33 (91.7)		22 (88)	27 (96.4)		10 (83.3)	39 (95.1)	
Divorced or widowed	4 (7.5)	1 (5.9)	3 (8.3)		3 (12)	1 (3.6)		2 (16.7)	2 (4.9)	
Relationship, n (%)				.46			.57			.13
Mother	38 (71.7)	12 (70.6)	26 (72.2)		18 (72)	20 (71.4)		10 (83.3)	28 (68.3)	
Father	13 (24.5)	4 (23.5)	9 (25)		7 (28)	6 (21.4)		1 (8.3)	12 (29.3)	
Other^c^	2 (3.8)	1 (5.9)	1 (2.8)		0 (0)	2 (7.2)		1 (8.3)	1 (2.4)	
Education level, n (%)				.37			.44			.09
High school or less	11 (20.8)	2 (11.8)	9 (25)		3 (12)	8 (28.6)		1 (8.3)	10 (24.4)	
Diploma	15 (28.3)	6 (35.3)	9 (25)		7 (28)	8 (28.6)		1 (8.3)	14 (34.1)	
Bachelor’s degree	24 (45.3)	9 (52.9)	15 (41.7)		13 (52)	11 (39.3)		9 (75)	15 (36.6)	
Postgraduate degree	3 (5.7)	0 (0)	3 (8.3)		2 (8)	1 (3.6)		1 (8.3)	2 (4.9)	
Occupation status, n (%)				.39			.91			.60
Employed	31 (58.5)	9 (52.9)	22 (61.1)		15 (60)	16 (57.1)		6 (50)	25 (61)	
Unemployed	12 (22.6)	3 (17.6)	9 (25)		5 (20)	7 (25)		4 (33.3)	8 (19.5)	
Retired	10 (18.9)	5 (29.4)	5 (13.9)		5 (20)	5 (17.9)		2 (16.7)	8 (19.5)	
Working hours per week, n (%)				.21			.61			.27
<20	8 (15.1)	1 (11.1)	7 (31.8)		3 (20)	5 (31.3)		3 (50)	5 (20)	
20‐40	20 (37.7)	6 (66.7)	14 (63.6)		11 (73.3)	9 (56.3)		3 (50)	17 (68)	
>40	3 (5.7)	2 (22.2)	1 (4.5)		1 (6.7)	2 (12.5)		0 (0)	3 (12)	
Economic security, n (%)				.09			.16			.04
Good	23 (43.4)	4 (23.5)	19 (52.8)		13 (52)	10 (35.7)		9 (75)	14 (34.1)	
Mediocre	26 (49.1)	12 (70.6)	14 (38.9)		9 (36)	17 (60.7)		3 (25)	23 (56.1)	
Poor	4 (7.5)	1 (5.9)	3 (8.3)		3 (12)	1 (3.6)		0 (0)	4 (9.8)	
Living space, n (%)				.46			.11			.04
House	26 (49.1)	9 (52.9)	17 (47.2)		15 (60)	11 (39.3)		9 (75)	17 (41.5)	
Apartment	27 (50.9)	18 (47.1)	19 (52.8)		10 (40)	17 (60.7)		3 (25)	24 (58.5)	

a Missing values: n=20.

b *P* values calculated using the chi-square test.

c Aunt and grandmother.

**Table 2.** Characteristics of the study sample—children with cerebral palsy (CP)^a^.

	Total sample (n=53)	Ease of Caregiving for Children			Child Engagement in Daily Life Measure			Satisfaction With Daily Occupations		
		Significant burden (n=17)	No significant burden (n=36)	*P* value^b^	Independent (n=25)	Dependent (n=28)	*P* value^b^	Yes (n=12)	No (n=41)	*P* value^b^
Sex, n (%)				.23			.22			.52
Male	11 (20.7)	2 (11.8)	9 (25)		7 (28)	4 (14.3)		2 (16.7)	9 (22)	
Female	42 (79.2)	15 (88.2)	27 (75)		18 (72)	24 (85.7)		10 (83.3)	32 (78)	
Age (years), mean (SD)	8.04 (2.75)	8.71 (3.62)	7.72 (2.22)	.23	7.52 (2.18)	8.50 (3.14)	.20	8.25 (2.52)	7.98 (2.84)	.77
BMI, (kg/m^2^)				.92			.45			.91
Underweight (<5th percentile)	13 (24.5)	5 (31.3)	8 (25)		4 (17.4)	9 (36)		2 (20)	11 (28.9)	
Normal (5th-85th percentile)	11 (20.8)	3 (18.8)	8 (25)		7 (30.4)	4 (16)		2 (20)	9 (23.7)	
Risk of overweight (85th-95th percentile)	4 (7.6)	1 (6.3)	3 (9.4)		2 (8.7)	2 (8)		1 (10)	3 (7.9)	
Overweight (>95th percentile)	20 (37.7)	7 (43.8)	13 (40.6)		10 (43.5)	10 (40)		5 (50)	15 (39.5)	
Diagnosis or topographic type, n (%)				.10			.06			.68
Spastic diplegic CP	7 (13.2)	2 (11.8)	5 (13.9)		2 (8)	5 (17.9)		2 (16.7)	5 (12.2)	
Spastic quadriplegic CP	5 (9.4)	4 (23.5)	1 (2.8)		0 (0)	5 (17.9)		0 (0)	5 (12.2)	
Spastic hemiplegic CP	6 (11.3)	3 (17.6)	3 (8.3)		2 (8)	4 (14.3)		1 (8.3)	5 (12.2)	
Ataxic CP	1 (1.9)	0 (0)	1 (2.8)		1 (4)	0 (0)		0 (0)	1 (2.4)	
Dyskinetic CP	34 (64.2)	8 (47.1)	26 (72.2)		20 (80)	14 (50)		9 (75)	25 (61)	
Gross Motor Function Classification System level, n (%)				.12			.49			.88
Level I	18 (34)	6 (35.3)	12 (33.3)		7 (28)	11 (39.3)		4 (33.3)	14 (34.1)	
Level II	10 (18.9)	2 (11.8)	8 (22.2)		4 (16)	6 (21.4)		2 (16.7)	8 (19.5)	
Level III	13 (24.5)	3 (17.6)	10 (27.8)		9 (36)	4 (14.3)		3 (25)	10 (24.4)	
Level IV	5 (9.4)	1 (5.9)	4 (11.1)		2 (8)	3 (10.7)		2 (16.7)	3 (7.3)	
Level V	7 (13.2)	5 (29.4)	2 (5.6)		3 (12)	4 (14.3)		1 (8.3)	6 (14.6)	
Functional level with hands, n (%)				.001			.001			.70
Independent	17 (32.1)	1 (5.9)	16 (44.4)		15 (60)	2 (7.1)		5 (41.7)	12 (29.3)	
Partially dependent	22 (41.5)	6 (35.3)	16 (44.4)		9 (36)	13 (46.4)		4 (33.3)	18 (43.9)	
Totally dependent	14 (26.4)	10 (58.8)	4 (11.1)		1 (4)	13 (46.4)		3 (25)	11 (26.8)	
Schooling, n (%)				.07			.004			.30
No	19 (35.8)	9 (53)	10 (27.7)		4 (16)	15 (53.6)		3 (25)	16 (39)	
Yes, regular	6 (11.3)	1 (5.8)	5 (13.8)		5 (20)	1 (3.6)		2 (17)	4 (10)	
Yes, private	28 (52.8)	7 (41.2)	21 (58.3)		16 (64)	12 (42.8)		7 (58.3)	21 (51.2)	
Number of people involved in childcare, n (%)				.03			.62			.55
≤3 persons	50 (94.3)	14 (82.4)	36 (100)		24 (96)	26 (92.9)		11 (91.7)	39 (95.1)	
>3 persons	3 (5.6)	3 (17.6)	0 (0)		1 (4)	12 (7.1)		1 (8.3)	2 (4.9)	
Total number of siblings, n (%)				.45			.40			.20
≤3 persons	32 (60.4)	11 (64.7)	21 (58.3)		17 (68)	15 (53.6)		9 (75)	23 (56.1)	
>3 persons	21 (39.6)	6 (35.3)	15 (41.7)		8 (32)	13 (46.4)		3 (25)	18 (43.9)	

a Missing values: n=20.

b *P* values calculated using the chi-square test.

The ECC mean raw score for all caregivers was 38.17 (SD 11.62; 95% CI 34.97‐41.37) and the mean scaled score was 48.78 (SD 16.18; 95% CI 44.33‐53.25), which showed that the entire sample had some degree of perceived burden. With further analysis using suggested cutoff scores (scaled score ≤40=substantial burden), 32.1% (17/53) of caregivers scored as having a significant perceived burden (Table 1). Two demographic factors were correlated with higher levels of perceived burden, the child’s functional level with hands (*P*=.001) and the number of people involved in childcare (*P*=.03; Table 2). Cross-tabulation of the questions of the self-care domain of the CEDLM with ECC cutoff scores (significant burden vs insignificant burden) showed that all questions were negatively correlated (ie, children of caregivers with perceived significant burden were more dependent; Table 3).

**Table 3.** Child Engagement in Daily Life Measure self-care (SC) domain items.

	Total sample, mean (SD)	Ease of Caregiving for Children			Satisfaction With Daily Occupations		
		Significant burden, mean (SD)	No significant burden, mean (SD)	*P* value	Yes, mean (SD)	No, mean (SD)	*P* value
SC1: feed self finger foods	3.64 (1.67)	2.29 (1.65)	4.28 (1.26)	.001	3.92 (1.68)	3.56 (1.68)	.39
SC2: feed self with a spoon or fork (solid food)	3.02 (1.57)	1.71 (1.16)	3.64 (1.36)	.001	3.67 (1.67)	2.83 (1.51)	.09
SC3: feed self with a spoon or fork (semisolid food)	3.17 (1.61)	1.88 (1.27)	3.78 (1.40)	.001	3.75 (1.71)	3.00 (1.57)	.14
SC4: feed self with a spoon or fork (liquid food)	2.83 (1.64)	1.41 (1.06)	3.50 (1.44)	.001	3.42 (1.56)	2.66 (1.65)	.15
SC5: drink from a closed cup or feeding bottle	3.30 (1.77)	2.18 (1.59)	3.83 (1.61)	.002	3.67 (1.83)	3.20 (1.76)	.35
SC6: drink from an open bottle or cup	3.55 (1.68)	2.18 (1.55)	4.19 (1.33)	.001	3.92 (1.68)	3.44 (1.69)	.24
SC7: dress upper body	2.30 (1.45)	1.41 (0.80)	2.72 (1.37)	.001	2.17 (1.47)	2.34 (1.33)	.63
SC8: undress upper body	2.47 (1.47)	1.47 (0.80)	2.94 (1.49)	.001	2.17 (1.47)	2.56 (1.48)	.40
SC9: dress lower body	2.32 (1.39)	1.29 (0.59)	2.81 (1.41)	.001	2.50 (1.62)	2.27 (1.34)	.72
SC10: undress lower body	2.53 (1.55)	1.35 (0.61)	3.08 (1.56)	.001	2.67 (1.83)	2.49 (1.49)	.84
SC11: wear shoes or socks or put on or take off an orthosis or brace	1.85 (1.03)	1.18 (0.39)	2.17 (1.08)	.001	1.92 (1.00)	1.83 (1.05)	.67
SC12: wash and dry hands	2.81 (1.65)	1.88 (1.45)	3.25 (1.57)	.003	3.17 (1.85)	2.71 (1.60)	.44
SC13: bathe or clean	2.30 (1.38)	1.47 (0.87)	2.69 (1.41)	.002	2.33 (1.67)	2.29 (1.31)	.88
SC14: body dry	2.25 (1.31)	1.35 (0.61)	2.67 (1.35)	.001	2.33 (1.50)	2.22 (1.28)	.87
SC15: combing hair	2.32 (1.44)	1.59 (1.23)	2.67 (1.41)	.004	2.42 (1.62)	2.29 (1.40)	.81
SC16: brush teeth	2.64 (1.62)	1.76 (1.25)	3.06 (1.62)	.006	3.00 (1.86)	2.54 (1.55)	.49
SC17: clean nose with a napkin	3.00 (1.60)	1.94 (1.35)	3.50 (1.48)	.001	3.25 (1.91)	2.93 (1.52)	.46
SC18: use the potty or toilet	2.30 (1.50)	1.35 (0.61)	2.75 (1.59)	.002	2.50 (1.73)	2.24 (1.45)	.68
Total	—^a^	23.21 (21.61)	51.32 (18.67)	—	44.92 (24.64)	41.53 (23.46)	—

a Not applicable.

The mean score for the self-care domain of the CEDLM was 48.60 (SD 23.09; 95% CI 42.24‐54.97), whereas the scaled score for this tool was 43.60 (SD 23.53; 95% CI 35.81‐48.79), which indicated that our sample scored near the lower end of the scale (Table 2). When suggested cutoff scores (scaled score ≥50.2=independent child) were used, the self-care domain of the CEDLM was positively correlated with the child’s functional level with hands (*P*=.001) and school enrollment (*P*=.004). Cross-tabulation with the questions of the ECC and self-care domain of the CEDLM cutoff scores revealed that all the questions but one (ECC 7: “to put on or take off an orthosis or brace”; *P*=.23) were negatively correlated (ie, the caregivers of independent children had less perceived burden; Table 4).

**Table 4.** Ease of Caregiving for Children (ECC) items.

		Child Engagement in Daily Life Measure (self-care domain)			Satisfaction With Daily Occupations		
	Total sample, mean (SD)	Independent, mean (SD)	Dependent, mean (SD)	*P* value	Yes, mean (SD)	No, mean (SD)	*P* value
ECC 1: to move at home and in the community?	2.74 (1.14)	3.24 (1.23)	2.29 (0.85)	.002	3.25 (1.22)	2.59 (1.10)	.08
ECC 2: to position for sleeping?	3.42 (1.21)	3.96 (1.10)	2.93 (1.12)	.002	4.00 (1.28)	3.24 (1.6)	.04
ECC 3: to position for feeding?	3.47 (1.20)	3.96 (1.06)	3.04 (1.17)	.006	3.83 (1.27)	3.37 (1.18)	.18
ECC 4: to position for bathing?	2.83 (1.72)	3.40 (1.12)	2.32 (0.98)	.001	3.33 (1.16)	2.68 (1.15)	.07
ECC 5: to position for playing?	3.21 (1.41)	3.80 (1.29)	2.68 (1.34)	.004	4.08 (1.31)	2.95 (1.36)	.01
ECC 6: to put on or take off clothing?	2.98 (1.16)	3.40 (1.00)	2.61 (1.19)	.01	3.58 (1.08)	2.80 (1.15)	.05
ECC 7: to put on or take off an orthosis or brace?	2.81 (1.09)	3.00 (1.00)	2.64 (1.16)	.23	3.42 (1.17)	2.63 (1.02)	.03
ECC 8: to bathe, clean, and tidy?	2.85 (1.11)	3.20 (1.16)	2.54 (0.99)	.03	3.33 (0.99)	2.71 (1.12)	.07
ECC 9: to use the potty or toilet, or for you to change his or her diapers?	2.87 (1.27)	3.44 (1.16)	2.36 (1.16)	.002	3.83 (1.12)	2.59 (1.18)	.003
ECC 10: to eat?	4.04 (1.16)	4.40 (1.00)	3.71 (1.21)	.03	4.58 (0.90)	3.88 (1.19)	.06
ECC 11: to drink?	4.13 (1.11)	4.64 (0.70)	3.68 (1.22)	.003	4.58 (0.90)	4.00 (1.14)	.10
ECC 12: to get in and out of a car, van, or bus?	2.83 (1.22)	3.36 (1.19)	2.36 (1.06)	.003	3.33 (1.30)	2.68 (1.17)	.12
Total	—^a^	56.46 (17.28)	41.93 (11.64)	—	56.96 (12.88)	46.39 (16.40)	—

a Not applicable.

Finally, the mean satisfaction score for SDO was 9.49 (SD 2.87; 95% CI 8.70‐10.28), indicating that the children participated in most daily occupations, and the mean satisfaction score with their activity level was 45.15 (SD 13.17; 95% CI 41.52‐48.78), which showed that the participants were somewhat satisfied with their daily occupations. Further analysis with established cutoff scores of the SDO (score ≥56=satisfied with daily occupations) showed that economic security and living space were negatively correlated with their SDO scores (ie, perceived mediocre or poor economic security and living in an apartment were linked with low SDO score; Table 1). Cross-tabulation with the questions of the ECC and SDO cutoff scores showed that some of the questions (questions 2, 5‐7, and 9) were negatively correlated with the perceived ease of care reported by the caregivers (ie, caregivers who had higher SDO scores had a perception of less caregiving burden; ECC 2: to position for sleeping; *P*=.04; ECC 5: to position for playing; *P*=.01; ECC 6: to put on or take off clothing; *P*=.05; ECC 7: to put on or take off an orthosis or brace; *P*=.03; ECC 9: to use the potty or toilet, or for you to change his or her diapers; *P*=.003; Table 4).

ECC scaled scores were positively correlated with the self-care domain of the CEDLM (*r*=0.64; *P*<.001) and the SDO (*r*=0.29; *P*=.03; Table 5). On the other hand, the self-care domain of the CEDLM was not correlated with the SDO score.

**Table 5.** Correlations among tools.

Comparison	Pearson coefficient (*r*)	*P* value
ECC^a^ vs self-care domain of CEDLM^b^	0.641	.001^c^
ECC vs SDO^d^	0.298	.03^e^
Self-care domain of CEDLM vs SDO	0.195	.16

a ECC: Ease of Caregiving for Children.

b CEDLM: Child Engagement in Daily Life Measure.

c Correlation is significant at the .01 level.

d SDO: Satisfaction With Daily Occupations.

e Correlation is significant at the .05 level.

## Discussion

### Principal Findings

To the best of our knowledge, this is the first study to explore the challenges faced by families of children aged 5 to 16 years with CP in Kuwait, including the perceived level of ease of care, caregivers' satisfaction with daily activities, and the associations of these outcomes with other possible factors. Analyses showed that caregivers of children with CP in Kuwait reported an average level of ease of care and an above-average satisfaction level with their performance of different life activities. The analyses also showed that the higher perceived ease of care was only associated with the child’s hand functional level and the number of people involved in childcare. The level of perceived ease of care reported in the current study was similar to that reported by caregivers in different regions of the world (ie, Poland and Turkey) [9,23]. This demonstrated that despite cultural and socioeconomic differences, the burden of caring for a child with CP was comparable [4]. On the other hand, the number of people involved in childcare being a significant factor can provide insights into a unique element of the culture in Kuwait and the surrounding region, where extended family and domestic workers [24] are involved in childcare and can affect the level of burden of care. This is a testament to the “village” families create to care for their children, which shows resilience and the coping measures taken to help care for children with CP.

Additionally, around half of the samples in this study scored at levels I and II of the GMFCS, which represents a higher functional capacity of the child. Nevertheless, the self-care domain of the CEDLM was toward the lower end of the scale as a total score and for individual items in our sample. This could be caused by a lack of focus on building independence (ie, performing self-care activities), which would reduce the number of tasks falling under the caregiver’s responsibility. Factors such as children’s hand functional level, their self-care abilities, and the number of people involved in childcare were found in previous studies to be variables that decreased the perceived level of caregiver burden for children with CP [9,10,23].

Contrary to expectations, this study did not find any significant difference in caregivers’ ease of care level according to the child’s BMI or CP subtype, which does not align with findings presented in the literature [9,23]. This finding may be explained by the characteristics of the children with CP, the majority of whom were at GMFCS levels I to III (41/53, 77.4%) while previous research was conducted with children at higher GMFCS levels. Additionally, the dyskinetic type of CP (34/53, 64.2%) was the most common type in our study; therefore, a lack of heterogeneity in the types of CP may have contributed to the findings [9,23].

A distinctive parameter that this study investigated was caregiver satisfaction with their daily life activities, and the literature suggests that the level of burden impacts caregivers’ quality of life [8,25]. Our findings demonstrated that caregivers were active participants in most of their children’s daily activities and were somewhat satisfied with their daily occupations. The current investigation also found that the higher the perceived ease of care, the more satisfied the caregivers were. This might be explained by an indirect association between the extensive care required when having a child with CP and the perceived burden of care that could hinder caregivers from being active members of the community [10].

A qualitative study conducted among parents of children with CP in Saudi Arabia revealed that caregivers expressed being under stress and experiencing challenges in navigating health and educational services [26]. These parents also emphasized the role of Islamic values expressed through religious practices in helping them cope with stress and perceived burden. The Kuwaiti population shares similar values to the Saudi Arabian population. Although it produced different data from the current study, that work showed trends similar to those exhibited in our study. These findings showed the need for further exploration of the population in the Arabian Peninsula region.

Given the differences identified in the present study compared with the published literature on caregiver burden among this patient population, it could conceivably be suggested that caring for children with CP in Kuwait should not rely on Western evidence alone. In a recent systematic review on the epidemiology of CP in Arabic-speaking countries, no articles referencing this population were found in Kuwait [27]. Given the scarcity of research in this region, normative data should be established for this population as it is crucial for different communities to understand their populations to tailor treatments to their communities’ specific needs.

As is the case with every scientific endeavor, this study is not without its limitations. The main limitation is the small sample size recruited, which also limited further investigation of some possible related factors, such as lower GMFCS levels or different CP subtypes. The nature of the cross-sectional study design limits the establishment of causal relationships or changes over time. Another limitation is self-report bias, which limits the interpretation of the results. The recruitment of participants from rehabilitation clinics may have introduced selection bias, which could have excluded caregivers of children with a high functional level and children with a very low functional level. Future studies should focus on adapting these classification systems for Arabic-speaking individuals to enable the use of these tools for upcoming research studies on children with CP and their caregivers. Further exploration of the individuals involved in caring for children with CP is warranted in future studies, especially differentiating between hired help and family members.

### Conclusions

CP is a lifelong health condition that requires special management and care throughout an individual’s life, impacting not only the individual with CP but also their family and primary caregivers. Caregivers might experience high levels of burden due to their assigned responsibilities, which could affect their life satisfaction. The preliminary perceived level of burden among caregivers of children with CP in Kuwait was correlated with caregivers’ daily activities satisfaction and the child’s self-care abilities. The findings of this study can inform practice in Kuwait and guide research advances in the region to improve care for caregivers of children with CP. Regular check-ins with caregivers and the establishment of caregiver support groups would help provide support sooner. Future studies with larger sample sizes and across multiple lifelong conditions would provide a clearer picture. Mixed methods strategies are encouraged in future studies to identify other contributing factors that may be unique to the region.

## Abbreviations

CEDLM: Child Engagement in Daily Life Measure
CP: cerebral palsy
ECC: Ease of Caregiving for Children
GMFCS: Gross Motor Function Classification System
ICC: intraclass correlation coefficient
SDO: Satisfaction With Daily Occupations

## References

[1] Sadowska M, Sarecka-Hujar B, Kopyta I (2020). Cerebral palsy: current opinions on definition, epidemiology, risk factors, classification and treatment options. Neuropsychiatr Dis Treat.

[2] Rosenbaum P, Paneth N, Leviton A (2007). A report: the definition and classification of cerebral palsy April 2006. Dev Med Child Neurol Suppl.

[3] Paul S, Nahar A, Bhagawati M, Kunwar AJ (2022). A review on recent advances of cerebral palsy. Oxid Med Cell Longev.

[4] Novak I, Hines M, Goldsmith S, Barclay R (2012). Clinical prognostic messages from a systematic review on cerebral palsy. Pediatrics.

[5] Dambi JM, Jelsma J, Mlambo T (2015). Caring for a child with cerebral palsy: the experience of Zimbabwean mothers. Afr J Disabil.

[6] Gabis LV, Tsubary NM, Leon O, Ashkenasi A, Shefer S (2015). Assessment of abilities and comorbidities in children with cerebral palsy. J Child Neurol.

[7] Freeman M, Stewart D, Cunningham CE, Gorter JW (2018). Information needs of young people with cerebral palsy and their families during the transition to adulthood: a scoping review. J Transit Med.

[8] Liu F, Shen Q, Huang M, Zhou H (2023). Factors associated with caregiver burden among family caregivers of children with cerebral palsy: a systematic review. BMJ Open.

[9] Gugała B (2021). Caregiver burden versus intensity of anxiety and depression symptoms in parents of children with cerebral palsy as well as factors potentially differentiating the level of burden: a cross-sectional study (Poland). BMJ Open.

[10] Keniş-Coşkun Ö, Atabay CE, Şekeroğlu A (2020). The relationship between caregiver burden and resilience and quality of life in a Turkish pediatric rehabilitation facility. J Pediatr Nurs.

[11] Muñoz-Marrón E, Redolar D, Boixadós M (2013). Burden on caregivers of children with cerebral palsy: predictors and related factors [Article in Spanish]. Univ Psychol.

[12] Park EY, Nam SJ (2019). Time burden of caring and depression among parents of individuals with cerebral palsy. Disabil Rehabil.

[13] Raina P, O’Donnell M, Rosenbaum P (2005). The health and well-being of caregivers of children with cerebral palsy. Pediatrics.

[14] Chaghazardi M, Janatolmakan M, Rezaeian S, Khatony A (2022). Care burden and associated factors in caregivers of children with cancer. Ital J Pediatr.

[15] Shah N, Badr H, Shah M (2012). Foreign live-in domestic workers as caretakers of older Kuwaiti men and women: socio-demographic and health correlates. Ageing Soc.

[16] Ward KD, Chiarello LA, Bartlett DJ, Palisano RJ, McCoy SW, Avery L (2014). Ease of Caregiving for Children: a measure of parent perceptions of the physical demands of caregiving for young children with cerebral palsy. Res Dev Disabil.

[17] Alghamdi MS, Chiarello LA, Abd-Elkafy EM, Palisano RJ, Orlin M, McCoy SW (2021). Cross-cultural adaptation of the Arabic version of Self-Care Domain of Child Engagement in Daily Life and Ease of Caregiving for Children measures. Res Dev Disabil.

[18] Alghamdi MS, Chiarello LA, Avery L, Palisano RJ (2020). Ease of caregiving for children: re-validation of psychometric properties of the measure for children with cerebral palsy up to 11 years of age. Dev Neurorehabil.

[19] Chiarello LA, Palisano RJ, McCoy SW (2014). Child Engagement in Daily Life: a measure of participation for young children with cerebral palsy. Disabil Rehabil.

[20] Manee F, Alotaibi N, Alobaidly F, Abu Tariah H, Hamed R, Eklund M (2015). The psychometric properties of the Arabic version of the Satisfaction with Daily Occupations. Br J Occup Ther.

[21] Eklund M (2004). Satisfaction with Daily Occupations: a tool for client evaluation in mental health care. Scand J Occup Ther.

[22] Hurvitz EA, Green LB, Hornyak JE, Khurana SR, Koch LG (2008). Body mass index measures in children with cerebral palsy related to gross motor function classification: a clinic-based study. Am J Phys Med Rehabil.

[23] Kaydok E, Solum S, Cinaroglu NS (2020). Comparison of the caregiver burden of the mothers of children with cerebral palsy and healthy children. Med Sci.

[24] Blaydes L (2023). Assessing the labor conditions of migrant domestic workers in the Arab Gulf states. ILR Rev.

[25] Barros AL, de Gutierrez GM, Barros AO, Santos MT (2019). Quality of life and burden of caregivers of children and adolescents with disabilities. Spec Care Dentist.

[26] Alqahtani A, Sahely A, Aldersey HM, Finlayson M, Macdonald D, Fakolade A (2025). Understanding the parental caregiving of children with cerebral palsy in Saudi Arabia: discovering the untold story. Int J Environ Res Public Health.

[27] Mushta SM, King C, Goldsmith S (2022). Epidemiology of cerebral palsy among children and adolescents in Arabic-speaking countries: a systematic review and meta-analysis. Brain Sci.

